# The Typhus Danger

**Published:** 1920-09-18

**Authors:** 


### The Typhus Danger.

### AN INDICTMENT OF THE LOUSE.

We hope there will be a good response to Mr. Balfour's strong appeal for £250,000 towards the £2,000,000 required by the League of Nations to combat the typhus epidemic in Poland and Russia. Apart from the disinterested motives of humanity, there is a practical reason why Great Britain should lend all the aid in its power to fighting this serious epidemic in the East of Europe. It may not appear that there is any immediate or direct danger to this country; but the danger would become real if the disease were to continue to spread westward unchecked, for even if, as is no doubt probable, proper precautionary measures would save us from any extensive visitation of the disease itself, we should still feel the very serious effects of the economic disturbances which must result if the vitality of half Europe were lowered by the general prevalence of the disease.

Fortunately, as *The Times* pointed out a few days ago, we know a good deal more about typhus now than we knew ten years ago, and we know that prompt and simple measures can be taken to deal with it effectively. It has been clearly demonstrated that the infection is carried by lice that are parasitic upon man. Mr. Bruce F. Cummings (known to the general public as Barbillion, the author of that extraordinary book, "The Journal of a Disappointed Man," but also an economic entomologist of great ability), wrote during the war a pamphlet on the louse and its relation to disease which was distributed to medical officers and unit commanders throughout the British Army. "Pediculosis," he wrote, "is always common in the poorer districts of most towns and villages, and has for years been rampant in the public elementary schools. In 1912 the medical officer of health in one of our northern counties reported that at the present rate of progress it would be several years before pediculosis among school children was reduced to ten or fifteen per cent., the main difficulties being constant re-infection in dirty homes and the absence of a safe and practicable remedy.

**THE PUBLIC WELL-BEING**—(*continued*).

The louse passes directly from person to person, and people of a cleanly habit are liable to become verminous through contact with verminous persons in houses, in railway carriages, with bedding in hotels and lodging-houses. The clothes louse always becomes abundant and troublesome wherever human beings—soldiers, for instance, on long campaigns—are gathered together in large numbers with infrequent opportunities for changing their clothing and washing. Pediculosis was rife among the Russians in the Crimean War, among our own soldiers during the South African War, and it was notoriously present among the troops in the Great War, though on the Western Front the sanitary conditions maintained by a first-class medical service were so excellent that typhus was a rare disease.

"It is now regarded as conclusively proved," said Mr. Cummings, "that exanthematic typhus, the disease which has devastated Serbia, is conveyed by the clothes louse, possibly by the head louse also, a fact which emphasises frequent occurrence of typhus among troops on active service. The prevention of typhus, therefore, is largely a question of exterminating lice. Both the clothes louse and the head louse are instrumental in spreading European relapsing fever, and also the relapsing fever of North Africa, by inoculation with the minute organisms known as spiroschaudinniæ. The method of infection is thought to be due to the irritation of the louse's bite, causing the patient to scratch and thus to crush the louse and at the same time to produce an abrasion of the skin. Scratching of irritant parts should, therefore, be avoided. This virus of typhus is probably conveyed in the same way. But it can also be transmitted by the simple puncture of the louse. The head louse also has come under suspicion of causing phlyctenular conjunctivitis, a disease of the eye frequent in infants and children. The spread of tubercle leprosy, plague, and other diseases has also been attributed to the agency of lice, but on insufficient evidence."

This indictment against the louse, however, is sufficiently severe. Where dirt is there is the louse, and where the louse is there is usually danger. In Great Britain typhus is now rare, though there have been severe outbreaks in Ireland, and too many cases occur there year after year. On the Eastern side of the continent of Europe all the conditions are favourable for a great extension of the disease, which seems to attack the strong male breadwinner of families first of all. At the present time the infectious activities of the louse are having more to do with the delay in the economic reconstruction of Europe than is pleasant to contemplate. It was reported a few days ago that 25,000 deaths from typhus are occurring monthly in Galicia and East Poland. This is considerably more than the number of British soldiers who were killed on the average each month during the Great War.

